# The relationship between foot arch measurements and walking parameters in children

**DOI:** 10.1186/s12887-016-0554-5

**Published:** 2016-01-23

**Authors:** Simone V. Gill, Sara Keimig, Damian Kelty-Stephen, Ya-Ching Hung, Jeremy M. DeSilva

**Affiliations:** Department of Occupational Therapy, Boston University, 635 Commonwealth Avenue, Boston, MA 02215 USA; Boston University Program in Rehabilitation Sciences, 635 Commonwealth Avenue, Boston, MA 02215 USA; Department of Medicine, Boston University Medical Center, 635 Commonwealth Avenue, Boston, MA 02215 USA; Department of Anthropology, Boston University, 635 Commonwealth Avenue, Boston, MA 02215 USA; Department of Psychology, Grinnell College, Grinnell, USA; Department of Family, Nutrition, and Exercise Sciences, Queens College, New York, USA; Department of Anthropology, Dartmouth College, Hanover, USA

**Keywords:** Gait, Children, Walking, Foot

## Abstract

**Background:**

Walking mechanics are influenced by body morphology. Foot arch height is one aspect of body morphology central to walking. However, generalizations about the relationship between arch height and walking are limited due to previous methodologies used for measuring the arch and the populations that have been studied. To gain the knowledge needed to support healthy gait in children and adults, we need to understand this relationship in unimpaired, typically developing children and adults using dynamic measures. The purpose of the current study was to examine the relationship between arch height and gait in a sample of healthy children and adults using dynamic measures.

**Methods:**

Data were collected from 638 participants (*n* = 254 children and *n* = 384 adults) at the Museum of Science, Boston (MOS) and from 18 4- to 8-year-olds at the Motor Development and Motor Control Laboratories. Digital footprints were used to calculate two arch indices: the Chippaux-Smirak (CSI) and the Keimig Indices (KI). The height of the navicular bone was measured. Gait parameters were captured with a mechanized gait carpet at the MOS and three-dimensional motion analyses and in-ground force plates in the Motor Development and Motor Control Laboratories.

**Results:**

Linear regression analyses on data from the MOS confirmed that as age increases, step length increases. With a linear mixed effect regression model, we found that individuals who took longer steps had higher arches as measured by the KI. However, this relationship was no longer significant when only adults were included in the model. A model restricted to children found that amongst this sample, those with higher CSI and higher KI values take longer relative step lengths. Data from the Motor Development and Motor Control Laboratories showed that both CSI and KI added to the prediction; children with lower anterior ground reaction forces had higher CSI and higher KI values. Arch height indices were correlated with navicular height.

**Conclusions:**

These results suggest that more than one measure of the arch may be needed elucidate the relationship between arch height and gait.

**Electronic supplementary material:**

The online version of this article (doi:10.1186/s12887-016-0554-5) contains supplementary material, which is available to authorized users.

## Background

Walking involves adapting movements to changes in local conditions [1]. For example, adults alter their gait to match the beat of an audio metronome [2] and to walk through moving apertures [3] or down slanted surfaces [4]. Similarly, children modify their gait to navigate over [5], around [6], and down [7] paths.

Changes in local conditions exemplify factors (e.g., environments) external to individuals. However, walking is also influenced by internal factors such as body morphology. Physical growth during childhood affects the kinematics [8] and kinetics [9] of walking. Changes in growth and body proportions from infancy through childhood render children less top heavy and more cylindrical. This change in growth allows for longer, straighter, and more coordinated steps [10] because the downward shift in center of mass increases stability [11]. Obesity also affects gait kinematics and kinetics; increased mass due to obesity is linked with decreasing velocity, step length, cadence, single limb support time and increasing double limb support time and step width in both children [12, 13] and adults [14, 15].

One aspect of body morphology central to walking has been linked to differences in the kinematics and kinetics of walking: the height of the foot arch. However, previous methodologies used for measuring the arch and the populations that have been studied limit generalizations that can be made about the relationship between arch height and walking. First, most studies examining arch height in relation to walking have used static measurements (i.e., during standing) of the arch [16, 17] with few having used dynamic measures (i.e., during walking) [18]. Second, our understanding of the arch height-walking relationship is largely based on adults with foot or gait pathologies [19–21]. To gain the knowledge needed to create interventions to support healthy gait in children and adults, we need to understand this relationship in unimpaired, typically developing children and adults using dynamic measures. Last, the anatomy of the foot allows for movements in multiple planes [22] and both the longitudinal (particularly the medial longitudinal arch) and transverse arches play a role. Yet, the measure of the foot arch is usually treated as a dichotomous rather than a continuous measure and may only capture either the mediolateral or anteroposterior curvatures of the foot.

The overall purpose of the current study was to examine the relationship between arch height and spatio-temporal gait measures in a sample of healthy children and adults using dynamic measures. First, we investigated the relationship between arch height indices and spatial walking parameters using dynamic rather than static methods [23]. Data collection took place at the Museum of Science, Boston, which provided access to a large number of participants across a wide range of ages. Second, we examined the arch height-gait relationship in a sample of 4- to 8-year old children in the laboratory where detailed gait analyses could be examined in a controlled, experimental setting. We chose this age range because children’s gait becomes adult-like between 5 and 7-years old [24]. We hypothesized that arch height would be predictive of gait kinematics and kinetics, including step length. This study is unique because it addresses how to capture the link between form (i.e., foot structure) and function (i.e., spatio-temporal walking parameters). Connecting form and function has clinical relevance because of the need to determine the nature of the relationship between form and function in order to investigate how intervening on one may influence the other.

## Method

### Participants

We first recruited and ran a total of 638 participants in the Living Laboratory® at the Museum of Science, Boston: 254 children ages 2 to 17 years old (*M* 9.13 years; *SD* = 3.26) and 384 adults from 18 to 80 years old (*M* 38.54 years; *SD* = 14.86). Inclusion criteria were that children and adults be able to walk independently, that children were typically developing, and that adults were not known to be pregnant (Table 1). The study and consent procedures were approved by the Boston University and Museum of Science, Boston’s Institutional Review Boards and conformed to the Declaration of Helsinki. Informed written and verbal consent was obtained from all participants before testing began. For children, informed consent was obtained from caregivers prior to their participation.

**Table 1:** Demographics and anthropometrics for children and adults from the Boston museum of science

	Age (years)	Sex	Weight (Kg)	Height (cm)	BMI (kg/m^2^)	Leg length (cm)	N
Children	9.13 (3.26)	F* 133; M* 121	35.14 (15.43)	134.29 (23.24)	18.36 (3.80)	70.28 (15.20)	254
Adults	38.54 (14.86)	F* 249; M* 135	71.48 (17.69)	167.66 (15.82)	25.09 (5.15)	90.52 (9.15)	384

Standard deviations are in parentheses

*F female, *M* male

Next, we recruited and tested 18 4- to 8-year-old children (*M* = 6.22 years; *SD* = 1.26) in the *Motor Development Laboratory* at Boston University and the *Motor Control Laboratory* at Queens College. Inclusion criteria were that children be able to walk independently (i.e., absent of any physical impairments that would preclude independent walking) and were typically developing (Table 2). The study and consent procedures were approved by the Boston University and Queens College Institutional Review Boards and conformed to the Declaration of Helsinki. Informed written and verbal consent was obtained from all participants before testing began. Caregivers provided informed consent prior to their children’s participation.

**Table 2:** Demographics and anthropometrics for children in the motor development and motor control laboratories

Age (years)	Sex	Weight (Kg)	Height (cm)	BMI (kg/m^2^)	Leg length (cm)	N
6.22 (1.26)	F* 8; M* 10	22.46 (4.34)	118.87 (8.99)	15.61 (1.32)	60.68 (6.81)	18

Standard deviations are in parentheses

*F female, *M* male

### Data acquisition

In the Museum of Science, we collected footfall recordings, digital pressure mat readings, and video recordings of participants.

#### Footfall recordings

Gait parameters were collected during walking sequences. The distance and timing of children’s and adults’ steps were measured with a pressure sensitive gait carpet (6.1-m long × 0.89 m wide) at a spatial resolution of 1.27 cm and a temporal resolution of 120 Hz (GAITRite Inc., Clifton, New Jersey, http://www.gaitrite.com). With the GAITRite software, spatial and temporal parameters were calculated via the x- and y-coordinates of the center of pressure of the heels and balls of the feet and the timing of foot onsets and offsets on the carpet. Specific parameters that were collected include: velocity, step length, step width, single limb support time, double limb support time, stance time, and step time.

#### Digital footprints

Digital footprints were gathered with a digital pressure mat to compute arch height indices. First, participants’ weight was obtained and used to calibrate measures for the digital pressure mat values. Second, dynamic plantar pressure was measured with a digital pressure mat (Tekscan Inc., South Boston, MA, www.tekscan.com). The mat (488 mm × 447 mm) collected data via 8448 sensing elements (4 sensel/cm^2^) at 185Hz. Tekscan software was used to locate peak pressure distributions from each sensor to create a digital footprint. Peak pressure foot profiles from the footprint were imported into ImageJ for processing.

**Fig. 1:**
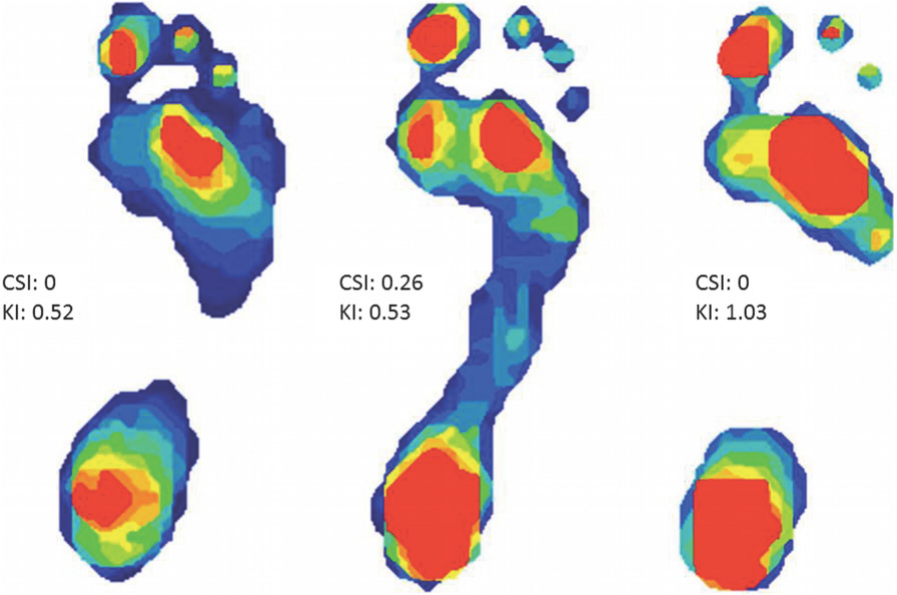
CSI and KI measurements. These are footprints from three individuals with similar CSI values, or even identical CSI measures (foot on far left and far right). Note that similar CSI values can originate from very different feet and that the KI captures some of those differences. For instance, even though the foot on the far left and far right have the same CSI values, the foot on the left exhibits more midfoot collapse and a lower KI value (similar to the middle foot), whereas the foot on the far right has a correspondingly higher KI value

Digital footprints were used to calculate two measures: the Chippaux-Smirak Index (CSI) and a new measure named the Keimig Index (KI); Fig. 1. The CSI [25] is the ratio between the smallest width of the mid-foot and the largest width of the metatarsal head area. A low CSI value is indicative of a higher arch whereas a high CSI value indicates that more of the midfoot is in contact with the ground and is thus characteristic of a lower arch. The second measure, the KI, quantifies the entire missing area of a footprint relative to the size of a toe-less footprint (see Additional file 1 for a fully outlined description of the KI). The KI quantifies the departure of the plantar surface of the foot from full contact with the ground surface, assuming the sagittal extrema of the heel and the balls of the feet. A higher KI value represents a higher arch, whereas a lower KI generally indicates a lower arched foot.

#### Video recordings

Participants were filmed from two perspectives. One camera captured the frontal view of participants. This camera was synchronized with the gait carpet software and was used to synchronize footfall and video recordings. A second camera filmed a zoomed-in sagittal view of participants’ feet at the end of walking sequences. This video was synchronized with the digital footprint data.

In the Motor Development and Motor Control Laboratories, we collected digital footprints using a digital pressure mat (Tekscan Inc., South Boston, MA, www.tekscan.com) and additional kinetics along with kinematics using equipment described below.

#### Force plates

Kinetic data were collected from two AMTI OR6-6 force platforms (each 46 × 50 cm). Data were processed and synchronized with the kinematic data at a rate of 1200 Hz with VICON Nexus 1.51.

#### Motion analysis

Three-dimensional kinematic data were collected with the whole body plug-in-gait model of VICON Nexus 1.51 with seven infrared cameras [26]. Collecting anthropometric measurements for each child prior to data acquisition ensured proper calibration. Reflective markers positioned bilaterally captured motion with x- (anterior/posterior), y- (medial/lateral), and z- (up/down) coordinates from the anterior and posterior portions of the anterior and posterior superior iliac spines, the lateral thighs, the knee joints, each tibia, the ankle joints, the heels, the big toes, and arch (i.e., at the height of the navicular tuberosity [27, 28]). All markers were digitized at a rate of 120 Hz with VICON Nexus 1.51. All digitized signals were processed with a low pass digital filter with a cutoff frequency of 6 Hz. Spatio-temporal variables of interest that were extracted were step length, step width, velocity, cadence, step time, stance time, single limb support time, and double limb support time.

### Procedure

In the Living Laboratory® at the Museum of Science, Boston, participants’ weight was obtained with a digital scale. Height was measured with a tape measure attached to a wall. Weight and height were used to calculate body mass index (BMI) in kg/m^2^. We measured the length of participants’ legs from the anterior superior iliac spine to the medial malleolus.

The gait carpet and digital pressure mat were placed abutting one another to create a continuous walking path approximately 6.5 m long. Participants stood at the very beginning of the walking path, and walked barefoot along the path for two trials. They were instructed to walk at a preferred walking speed without stopping until the end of the path. Trials were processed using GAITRite software. Both trials were averaged for each individual for statistical analyses. Methods used for running walking trials and processing walking data for children were conducted the same as in previous studies [7, 29], which ensured useable trials for analyses.

In the Motor Development and Motor Control Laboratories, after an auditory go signal, children walked at a self-selected pace on a 6.5 m-long path. The digital pressure mat was placed over the two AMTI OR6-6 force platforms (each 46 × 50 cm) to collect simultaneous digital footprint and force measures as children walked. Trials ended when children walked to a stop line at the end of the walking path. Children received 3 practice trials to become familiarized with the task. They walked for a total of 10 trials. Averages for all trials were computed per child for further analysis. Data collection and processing techniques for children were identical to procedures used in other child labs to ensure useable data [7, 29].

### Statistical analyses

SPSS 20.0 statistical software was used to complete all analyses. The results were presented as means (*M*) and standard deviations (*SD*) and/or counts as appropriate. With data collected at the Museum of Science, we aimed to model the effects of arch measures on differences in spatial gait parameters (i.e., step length and step width). First, linear regression analyses were run to confirm differences in step length and step width across age. Parameters were normalized by leg length. To make predictions, we used a linear mixed effect (LME) regression to model the effects of arch measures on differences in measured step length across ages [30]. Further, we included BMI as a covariate because BMI is known to reduce step length and increase step width [31, 32]. We sought to test the combined effects of KI and CSI on step length across age in children, above and beyond their effects on step width. With data collected in the Motor Development and Motor Control Laboratories, separate Pearson’s correlations were run on navicular height and CSI as well as navicular height and KI to investigate the association between structural and footprint measures of arch height. Kinematic gait parameters included step length, step width, velocity, cadence, step time, stance time, single and double limb support times. Kinetic gait parameters were ground reaction forces in the anterior/posterior, medial/lateral, and vertical directions normalized by weight during single limb support time at maximum knee height for the contralateral leg. Separate multiple regression analyses were conducted using the CSI and KI to predict gait parameters. Data for the CSI, KI, and ground reaction forces are reported for the left foot because our analyses showed no differences in measures for the left and right feet. Statistical significance was set at 0.05 (two-tailed) with Bonferroni adjustments for follow up comparisons.

## Results

### Available data

At the Museum of Science, data from 254 children and 384 adults were collected. Due to equipment failure, data were lost for footfall recordings (*n* = 4 children, *n* = 2 adults) and digital footprints (*n* = 17 children, *n* = 17 adults). Therefore, 233 children and 365 adults had footfall recordings and digital footprints available for analyses. Spatial gait parameters and arch height measures for children and adults are in Table 3. With data collected in the Motor Development and Motor Control Laboratories, data for all 18 children were available for analyses (Tables 4 and 5).

**Table 3:** Spatial gait parameters and arch height measures (i.e., CSI and KI) for children and adults from the Boston museum of science

	Step length (cm)	Step width (cm)	CSI	KI	N
Children	56.11 (10.07)	8.40 (2.70)	0.14 (0.15)	0.59 (0.26)	233
Adults	64.52 (6.82)	9.94 (3.07)	0.20 (0.15)	0.49 (0.20)	365

Standard deviations are in parentheses

**Table 4:** Spatial gait parameters for children from the motor development and motor control laboratories

Step length (cm)	Step width (cm)	Velocity (cm/s)	Cadence (steps/min)	Step time (msec)	Stance time (msec)	Single limb support time (msec)	Double limb support time (msec)	N
42.83 (4.86)	7.73 (1.44)	107.90 (10.98)	135.57 (10.54)	399.13 (28.15)	413.61 (31.60)	288.16 (17.58)	123.24 (20.89)	18

Standard deviations are in parentheses

**Table 5:** Arch height measures (i.e., CSI and KI) and left ground reaction forces normalized by weight in the anterior/posterior (Left Norm A/P GRF), medial/lateral (Left Norm M/L GRF), and vertical (Left Norm Vertical GRF) directions from the Motor Development and Motor Control Laboratories

Left CSI	Left KI	Left Norm A/P GRF (N/kg)	Left Norm M/L GRF (N/kg)	Left Norm Vertical GRF (N/kg)	N
0.05 (0.06)	0.58 (0.26)	2.00 (1.04)	1.37 (0.75)	6.31 (1.79)	18

Note that CSI, KI, and ground reaction forces are shown for the left foot. Standard deviations are in parentheses

### Model predictions for effects of arch height on gait

**Fig. 2:**
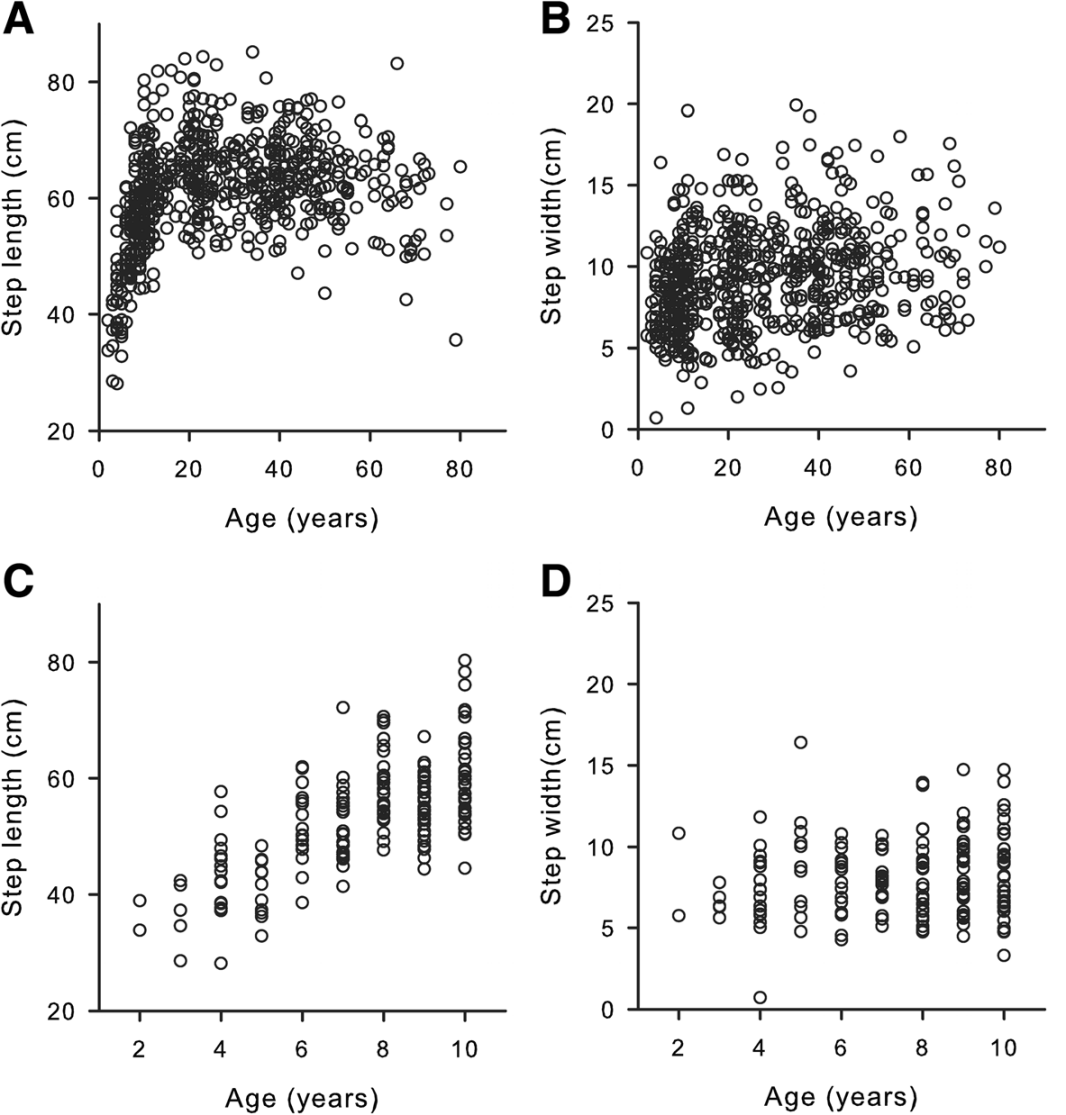
Step length (**a**) and step width (**b**) by age. Each circle represents one participant’s average. Notice the steep slope in children who rapidly achieve adult step length, which remains constant through much of adulthood and declines later in life. Figure 2c and d highlight children’s step length (**c**) and step width (**d**) from 2 to 10 years old in our sample

Using the data collected at the Museum of Science, we first confirmed changes in step length (Fig. 2a) and step width (Fig. 2b) that occur across age, particularly during early childhood (Fig. 2c–d); linear regression analyses confirmed that as age increases, both step length (*F*(1630) = 195.46, *p* < .001, *R*^*2*^ = .24) and step width (*F*(1630) = 7.93, *p* < .01, *R*^*2*^ = .01) increase. Although step width typically decreases within the first six months of walking [29, 33], our older sample shows increases and a similar range in step width as demonstrated in previous studies [29].

**Fig. 3:**
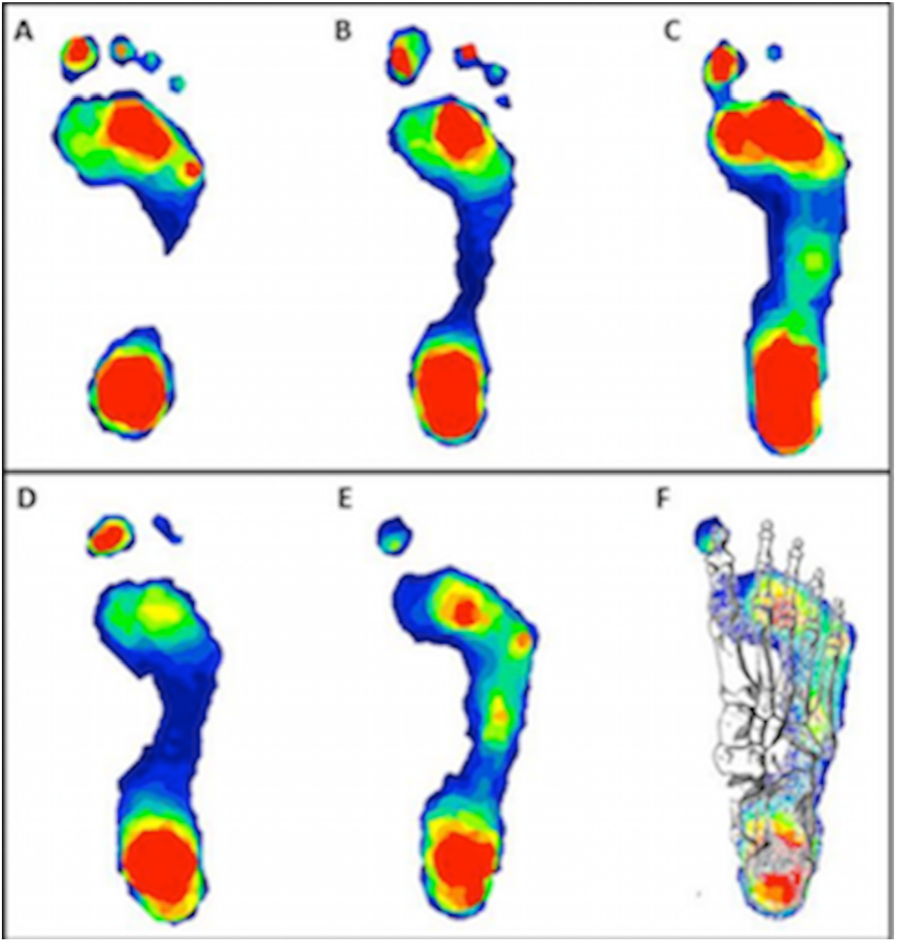
Digital footprints. Top panel: footprints of three children with different arch heights (**a**, **b**, **c**) as measured by both the CSI and KI with the highest arch to the left (**a**) and a flatfoot to the right (**c**). Bottom panel: footprints of two children with similarly developed lateral arches (same CSI values) but distinct shapes to the medial arch (different KI values) (**d**, **e**, **f**). Our findings indicate that for a given CSI, those with a higher KI (to the right) (**f**) take longer step lengths than those with lower KI (to the left) (**d**). Bottom right (**f**): the plantar view of a foot skeleton has been superimposed on the child’s footprint to indicate which bones (talus, navicular, medial cuneiform, and first metatarsal) contribute to the high medial arch

**Fig. 4:**
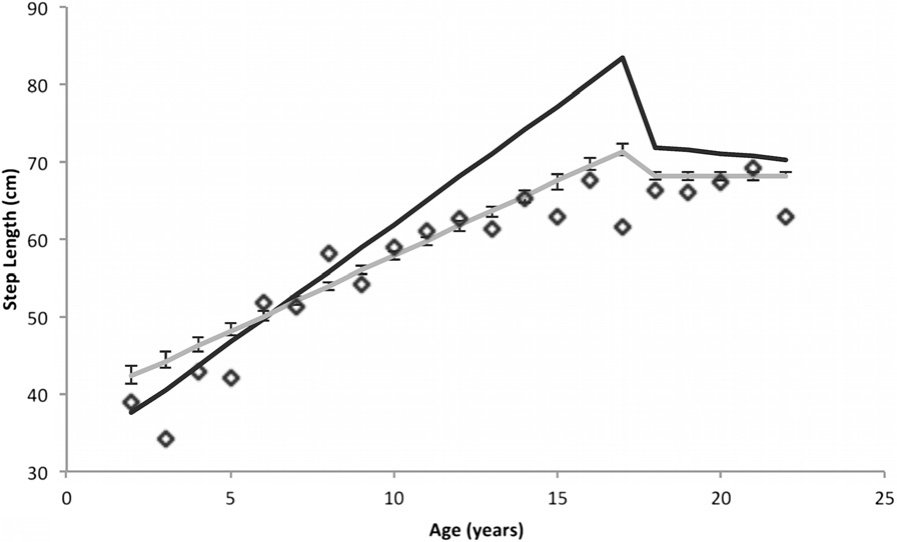
Plot of step length by age in years. The diamonds indicate the average measured step length for participants of each age in years. The black line depicts the model predictions for estimated coefficients with high values (i.e., 3^rd^ quartile) of KI and CSI. The grey line depicts the average model predictions for all other combinations of high and low values of KI and CSI, for high and low values of BMI as well. Error bars around the grey curve indicate two standard errors around these average predictions for all but the High-KI/High-CSI case. The figure shows that the High-KI/High-CSI line (black line) captures the growth of step length over the earlier years of childhood (age 2 to, roughly, 8 or 9) better than the average model predictions depicting other combinations of KI and CSI (grey line). These differences suggest that early childhood growth of step length may be promoted by the combination of a high KI and a high CSI

Next, we used CSI and KI to model predictions about gait. Gait variables were entered into an LME regression model. We found that individuals who took longer steps had higher medial longitudinal arches as measured by the KI. However, this relationship was no longer statistically significant when only the adults were included in the model. Furthermore, the model was strengthened when both measures of arch height (CSI and KI) were included, suggesting that they were measuring slightly different aspects of foot anatomy. For instance, although some individuals may have similar CSI values due to similarly developed lateral longitudinal arches, they may have differently shaped medial longitudinal arches, which would result in different KI values (Fig. 3). Also, particular values of CSI and KI led to better model predictions for walking patterns. Specifically, the best model to predict step length included values with high CSI and high KI values. A model restricted to children found an interaction effect suggesting that those with both higher CSI and higher KI values take longer relative step lengths (*B* = 10.50, *SE* = 2.93, *p* < .001), potentially meaning that lower transverse and higher medial longitudinal arches were associated with longer steps (Fig. 4).

### Relationship of navicular height with CSI and KI

Using the data collected in the Motor Development and Motor Control Laboratories, we aimed to confirm that CSI and KI were related to a commonly used structural indicator of arch height: the height of the navicular bone. Navicular height was correlated with both the CSI (*r* (18) = −0.49, *p* < .05) and KI (*r* (18) = 0.54, *p* < .05) measurements; children with higher navicular measures had lower CSI and higher KI measures.

### Model predictions of gait from CSI and KI

Multiple linear regression analyses on the data collected in the Motor Development and Motor Control Laboratories showed that using footprint measures predicted measures of children’s gait. Arch height indices predicted step length (*F*(2,15) = 4.74, *p* < .05, *R*^*2*^ = .39) with CSI adding significantly to the prediction (*p* < .02). No other kinematic gait parameters were predicted by arch height indices (all *p*s > .05) presumably because our arch index measures had stronger predictive abilities for spatial rather than temporal gait parameters. Analyses also revealed that footprint measures were predictive of kinetic measures. Arch height indices predicted anterior/posterior force (*F*(2,15) = 5.58, *p* < .05, *R*^*2*^ = .43). Both CSI and KI significantly added to the prediction (all *p*s < .02); children with lower anterior ground reaction forces had higher CSI and higher KI values (Tables 4 and 5).

## Discussion

The purpose of this study was to examine the relationship between the height of the foot arch and walking in children and adults. Our findings showed that CSI and KI, which both correlate with navicular height, predicted gait kinematics and kinetics for children. Specifically, higher CSI and KI predicted longer steps and lower anterior ground reactions forces. These predictions were only true for children and required both the CSI and the KI.

Our results support efforts to treat the arch as a continuous rather than categorical feature of the foot. Previous work has often categorized variation around some central “normal” as dichotomous pathologies of the foot such that these foot arches were presumed to be either flat or high. Treating the arch as categorical only considers the shape of the arch in the sagittal plane, but our bodies move in multiple planes and rely on the structure of the arch while doing so particularly in light of foot structure [34].

Our data suggest that each arch height index provided unique information for children. The CSI appears to quantify the arch of the foot mediolaterally and may be capturing the general height and morphology of the transverse arch. The KI attempts to measure the medial longitudinal arch arch proximodistally. A weak arch that collapses medially may still have an “arched” foot using the CSI, but would have a much lower KI.

These findings suggest that a particular arch profile for children predicts gait kinematics and kinetics. That is, if CSI and KI are in fact capturing the anatomy we have hypothesized, gait kinematics and kinetics were predicted by relatively low transverse (i.e., high CSI), but high medial longitudinal arches (i.e., high KI) in children. On average, children begin walking at 12 months old, but continue to refine their walking until 5- to 7-years old [29]. Therefore, although they demonstrate gains in walking skill (e.g., increased step length), they may still require stability as their walking skill continues to improve. The transverse and medial longitudinal arches may be serving this role for children; high medial longitudinal arches help execute longer step lengths while low transverse arches may help maintain stability. In addition, the disappearance of children’s “fat pads” [35–37] in the feet may not only influence movement in the sagittal plane to lift the arch off of the ground. As the fat pad diminishes foot contact with the ground from the medial to the lateral aspect of the foot, loss of the fat pads could also affect frontal plane motion.

Our results have practical implications for aiding improvements in children’s walking patterns. Understanding walking in typically developing children can help in treatment with atypical development. Specifically, in typical development, children’s steps begin as short and uncoordinated and develop into longer, more coordinated steps [29]. Our results suggest that the shape of the foot is related to walking patterns (i.e., high CSI and high KI are associated with longer step lengths). Thus, in children who tend to take shorter steps due to difficulties with walking, it may be beneficial to facilitate taking longer steps by using orthotics that enable lower medial longitudinal arches and high transverse arches.

## Conclusions

In summary, these results suggest that more than one measure of the arch may be needed to elucidate the relationship between arch height and gait, particularly for children. Results suggest that the shape of the foot is related to spatio-temporal walking patterns.

### Limitations

One limitation includes finding modest relationships between navicular height and CSI and KI. However, our results highlight new information regarding the relationship between arch height and walking, particularly in children. A second limitation is that we did not capture non-weight bearing arch height in participants. However, the focus of the current study was on foot structure during a weight bearing activity. Third, our finding that children with high CSI and high KI values had longer step lengths and lower anterior ground reaction forces may appear to be unusual. This finding may be due to when we sampled our ground reaction force measurements: at midstance. Taking our force measurement at midstance allowed us to be consistent with the time at which digital footprints were used to calculate the CSI and KI: during full weight bearing at midstance.

## Additional file

Additional file 1:
**How to Calculate the Keimig Index (KI).** (DOCX 377 kb)
