# Supplementary material for: The relationship between foot arch measurements and walking parameters in children
Source: BMC Pediatr. 2016 Jan 23;16:15. doi: 10.1186/s12887-016-0554-5 (PMC4724397; doi:10.1186/s12887-016-0554-5)
Supplement: Additional file 1: — How to Calculate the Keimig Index (KI). (DOCX 377 kb) [file 12887_2016_554_MOESM1_ESM.docx]

Additional file1:

**How to Calculate the Keimig Index (KI)**

1) If using the Tekscan software:

Make sure that you are viewing peak pressures (a). Then, to make copying and measuring the image easier, make the image all one color by using the calibration

tool (b). Set the calibration tool to 1.

b


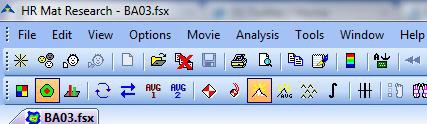

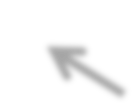

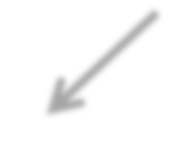

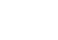

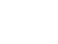


a


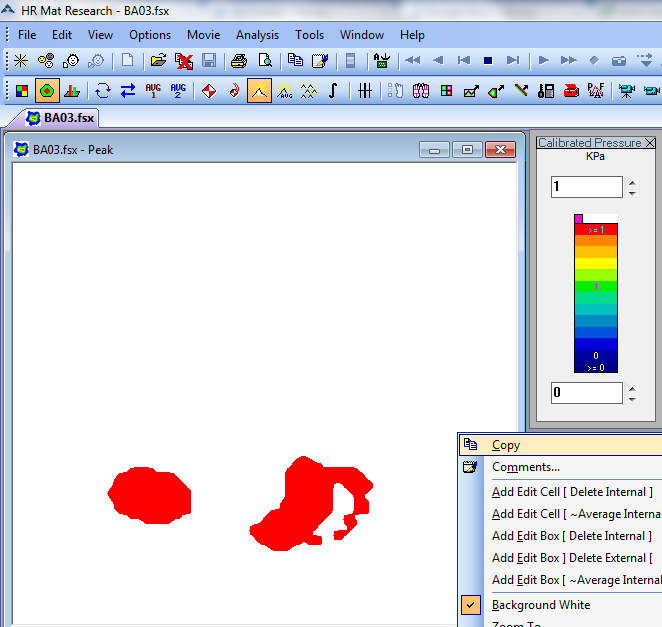


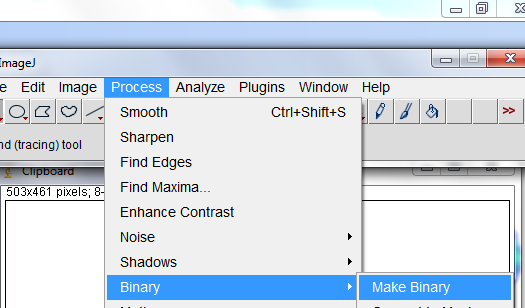
2) Open the ImageJ software, and paste the image. Go to Process > Binary > Make Binary to make the image black, and able to be measured.

3) Click the Freehand Selections tool and use it to select the toe area to remove. Once selected, click Edit > Clear. If you are going to save the image, it is a good idea to save it now, and as a PNG file.


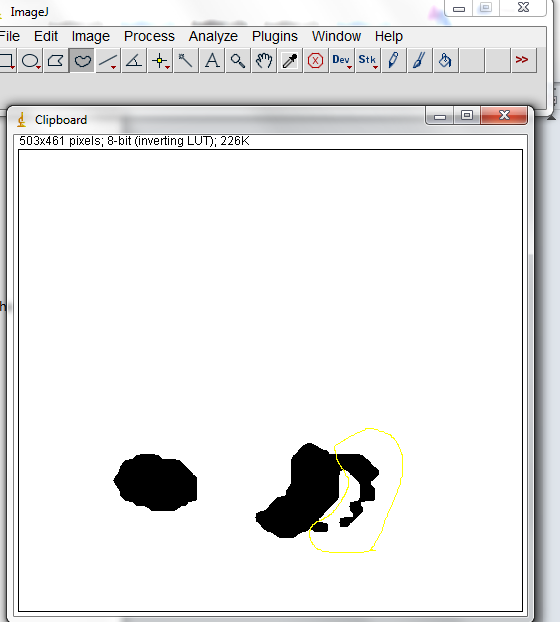

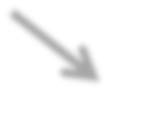


4) Use the Wand (tracing) Tool to find the area of the actual footprint. Click the foot (or each part of the foot) and make sure the area is highlighted in yellow. Then press Ctrl+M. Use the value under Area for the total footprint area. In this example, you will need to do this separately for each part and add the areas together (or press and hold Shift when using the Wand tool.


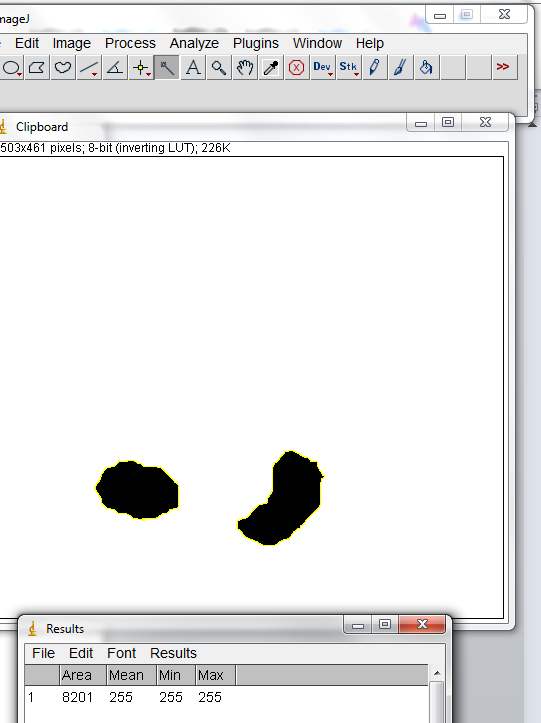

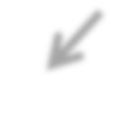


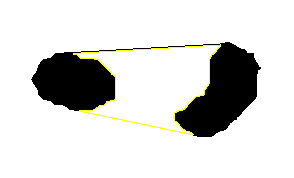
5) Use the straight line tool to make a line connecting the two most medial points of the footprint. After the line has been created, press Ctrl+D to actually draw the line on the image. Repeat this process for the lateral side of the foot. Then, use the Wand Tool to find the area. Sometimes you may need to try click in a few different areas. Try in the middle of the missing arch arch and along a few of the borders until the area you are attempting to trace has been selected in yellow. Then, press Ctrl+M. The most recent value under Area is the area of the missing midfoot region. In this example, because the midfoot is entirely missing, there is only one area to measure. In most footprints, you will need to find an area on the medial side and the lateral side, and add the values together. Finally, divide the total missing area (both medial and lateral sides) by the total area of the footprint to calculate the Keimig Index.
